# Slide scanner-based microscopy image datasets of fossil diatoms from Southern Ocean surface sediments

**DOI:** 10.1016/j.dib.2025.112404

**Published:** 2025-12-19

**Authors:** Saki Ishino, Takuya Itaki

**Affiliations:** a Geological Survey of Japan, National Institute of Advanced Industrial Science and Technology, 1-1-1 Higashi, Tsukuba, Ibaraki 305-8567, Japan; b Estuary Research Center, Shimane University, 1060 Nishikawatsu-cho, Matsue-shi, Shimane 690-8504, Japan

**Keywords:** *Eucampia antarctica*, Paleoenvironmental indicator, Object detection, Light microscopy, Biogeography

## Abstract

This dataset consists mainly of scanned images of permanent slides of surface sediments for the observation of fossil diatoms. The sediment samples were collected from a wide range of locations across the Southern Ocean. The permanent slides were prepared using standard processing, including hydrogen peroxide treatment and the pipette method. The image data include whole-coverslip scans (in the original format of Hamamatsu Photonics K.K.) and tile images (in JPEG format) segmented into 552 μm squares from the whole scanned area. All images have a resolution of 0.23 μm/pixel, which is sufficient for counting marine diatoms. The accompanying annotations (YOLO format) are specifically designed for detecting *Eucampia antarctica* (Castracane) Mangin, a key paleoenvironmental indicator. The tile images and annotations can be used to train and test object detection models for *Eucampia antarctica*, which can then be applied to morphometric analyses. The broad applicability of this dataset supports biogeographical and paleoenvironmental research through image-based analyses of fossil diatoms.

Specifications Table

**Table:** 

Subject	Earth & Environmental Sciences
Specific subject area	Biogeography, Geology, Paleoceanography, Paleontology, Computer vision
Type of data	Digital microscopy slide images (ndpi and jpg), Annotations for YOLO format (txt), Trained YOLO models (pt), Information tables (csv)
Data collection	The permanent slides were prepared from hydrogen peroxide–treated surface (0–2 cm) sediment samples collected from 18 sites using a grab sampler, a gravity corer, or a multiple corer. Each virtual slide capturing an entire coverslip area was acquired by NanoZoomer SQ (Hamamatsu Photonics K.K.) and preserved in ndpi format. The virtual slides were divided into tile images in jpg format with the dedicated software. The virtual slide scanning settings associated with original permanent slides and the site locations of the samples used were summarized in csv files. The YOLO-format annotations for a specific fossil diatom taxon were prepared using the Visual Object Tagging Tool (VOTT, v2.2.0; Microsoft).
Data source location	1. Site: KH19–6-MC10. Institution: Atmosphere and Ocean Research Institute, The University of Tokyo Location: 60°53.67′S, 17°28.512′W. 2. Site: JARE59-CD. Institution: National Institute of Polar Research (NIPR). Location: 68°19.368′S, 68°55.368′E. 3. Site: JARE61-St. 83-LGC. Institution: NIPR. Location: 67°45.21′S, 118°15.078′E. 4. Site: JARE61-St. 12B-LGC. Institution: NIPR. Location: 67°13.578′S, 117°6.288′E. 5. Site: G404. Institution: Japan National Oil Corporation (JNOC). Location: 66°31.158′S, 108°0.06′E. 6. Site: G501. Institution: JNOC. Location: 67°50.01′S, 74°35.286′E. 7. Site: G503. Institution: JNOC. Location: 68°41.964′S, 72°35.97′E. 8. Site: GC1004. Institution: JNOC. Location: 68°19.368′S, 68°55.368′E. 9. Site: GC1006. Institution: JNOC. Location: 69°51.318′S, 72°18.078′E. 10. Site: GC1509. Institution: JNOC. Location: 64°24.282′S, 118°10.272′E. 11. Site: GC1903. Institution: JNOC. Location: 61°28.638′S, 95°2.724′E. 12. Site: GC1904. Institution: JNOC. Location: 65°54.354′S, 80°22.914′E. 13. Site: GC1905. Institution: JNOC. Location: 65°53.928′S, 85°10.932′E. 14. Site: GC2001. Institution: JNOC. Location: 63°43.59′S, 67°21.162′E. 15. Site: GC2002. Institution: JNOC. Location: 60°29.286′S, 64°29.46′E. 16. Site: GC2003. Institution: JNOC. Location: 63°57.33′S, 74°8.754′E. 17. Site: GC2006. Institution: JNOC. Location: 67°59.928′S, 73°4.356′E. 18. Site: GC2009. Institution: JNOC. Location: 66°54.924′S, 75°4.326′E. All surface sediment samples and permanent slides are stored in the Geological Survey of Japan, Tsukuba, Ibaraki, Japan.
Data accessibility	Repository name: Mendeley Data Data identification number: DOI:10.17632/457×9c7rp3.2 Direct URL to data: https://data.mendeley.com/datasets/457×9c7rp3/3
Related research article	S. Ishino, T. Itaki, M. Fukuda. Deep learning object detection for fossil diatom counting: assessing the impact of fossil preservation and intraspecific morphological variation. Marine Micropaleontology, 2025, 102,519. https://doi.org/10.1016/j.marmicro.2025.102519

## 1 Value of the Data

**Fig. 1:**
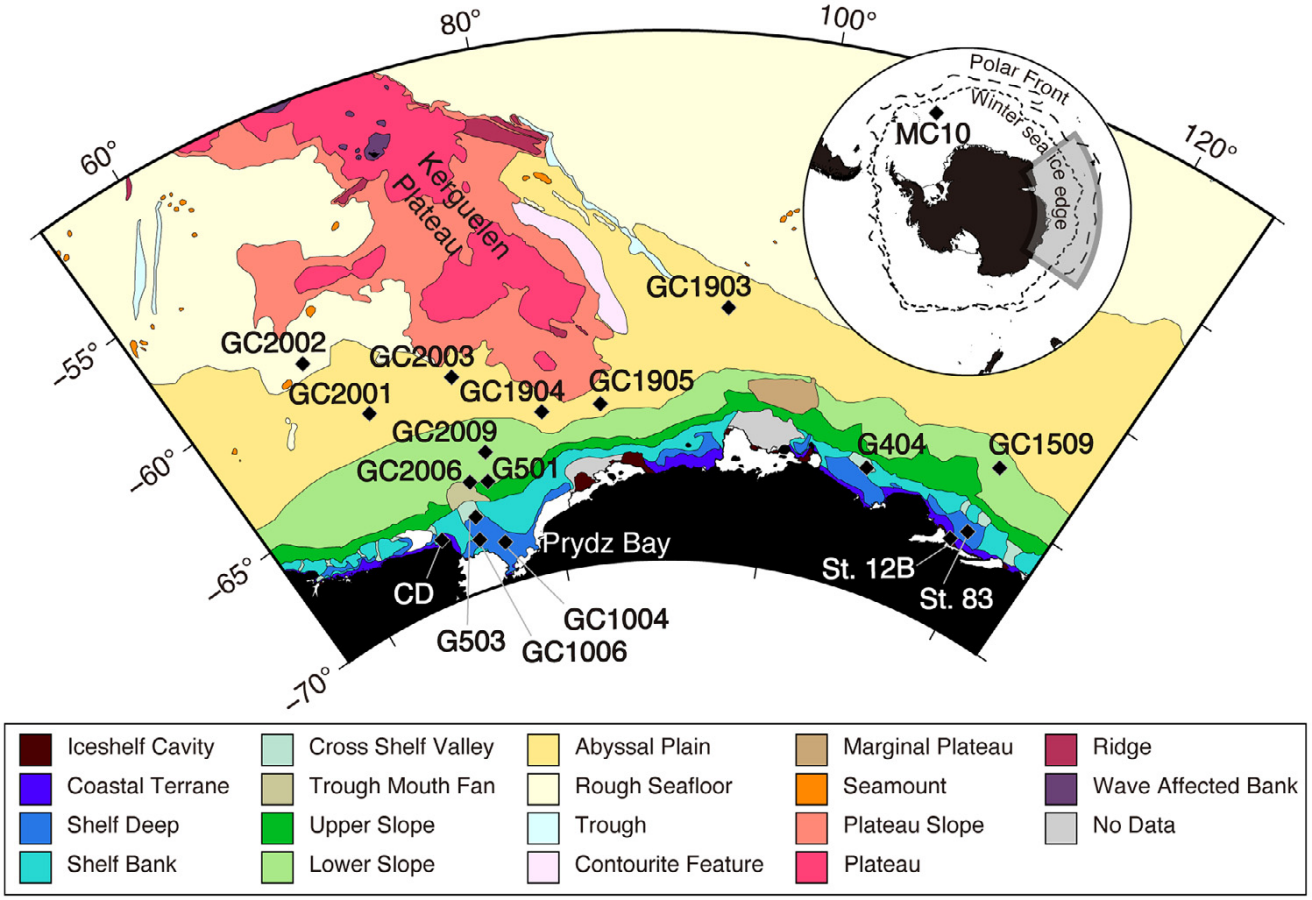
Map of sampling sites for the surface sediments used in this dataset. Plotted geomorphic features, which may reflect variations in sedimentary and oceanographic environments, are modified from Post et al. [4]. The positions of the winter sea-ice edge and the Polar Front are mapped after Smith and Jacka [5] and Orsi et al. [6], respectively.
• The accompanying annotations and trained YOLO models are designed to detect *E. antarctica*, allowing their use as training and testing data for object detection tasks. The developed models can extract cropped images of this species from large sets of newly acquired microscopy images in a significantly shorter time than manual processing. This approach reduces the effort required for morphological analyses of downcore and geographically diverse samples.
• The image dataset itself can be reused for image-based biogeographical and morphological analyses of various Southern Ocean diatom taxa beyond *E. antarctica*. The dataset will also support future deep learning-based model development for fossil diatoms by providing training data for both classification and object detection tasks.

• Analyses of fossil diatom assemblages and their morphometric characteristics have provided valuable proxies for paleoenvironmental reconstructions using sediment cores. In the Southern Ocean, *Eucampia antarctica* (Castracane) Mangin plays a key role in reconstructing past changes in sea-ice concentration and sea surface temperature [1,2].
• This dataset contains scanned images of permanent diatom slides prepared from surface sediments collected across the Southern Ocean. The geographically diverse samples (Fig. 1) help biogeographical investigations based on image analysis and the development of more spatially comprehensive paleo-proxies [3].

## 2 Background

Investigations of fossil diatom biogeography, including analyses of morphological and assemblage features, have significantly contributed to establishing paleoenvironmental indicators for sediment core analyses [[1], [7], [8]]. However, traditional manual counting approaches remain time-consuming for taxonomists, posing a significant limitation for processing large numbers of samples within a short timeframe. The integration of image-based and deep-learning approaches helps address this challenge. This dataset includes microscopy images from a wide range of geomorphological settings and water depth across the Southern Ocean and covers the major morphological variations of *Eucampia antarctica*, a key paleoenvironmental indicator [3]. Furthermore, as supported by related research [3], object detection models trained on these datasets for extracting this species maintain high performance across these sites, regardless of morphological diversity. This dataset was constructed from standard permanent slides used for routine diatom observations; therefore the models can be applied directly to both archived and newly acquired samples without additional sample treatment. Making the associated methods, microscopy images, and trained models publicly available will facilitate future expansion of similar datasets and their applications in paleoenvironmental research, for both diatom taxonomists and researchers in other disciplines who work with fossil diatom data.

## 3 Data Description

This work focuses on providing the transmitted light microscopy image sets of permanent slides from the Southern Ocean and on developing methods that support faster and more accurate counting of a specific fossil diatom taxon (*E. antarctica*). The dataset consists mainly of virtual slides, tile images, annotations, and trained models.

**Fig. 2:**
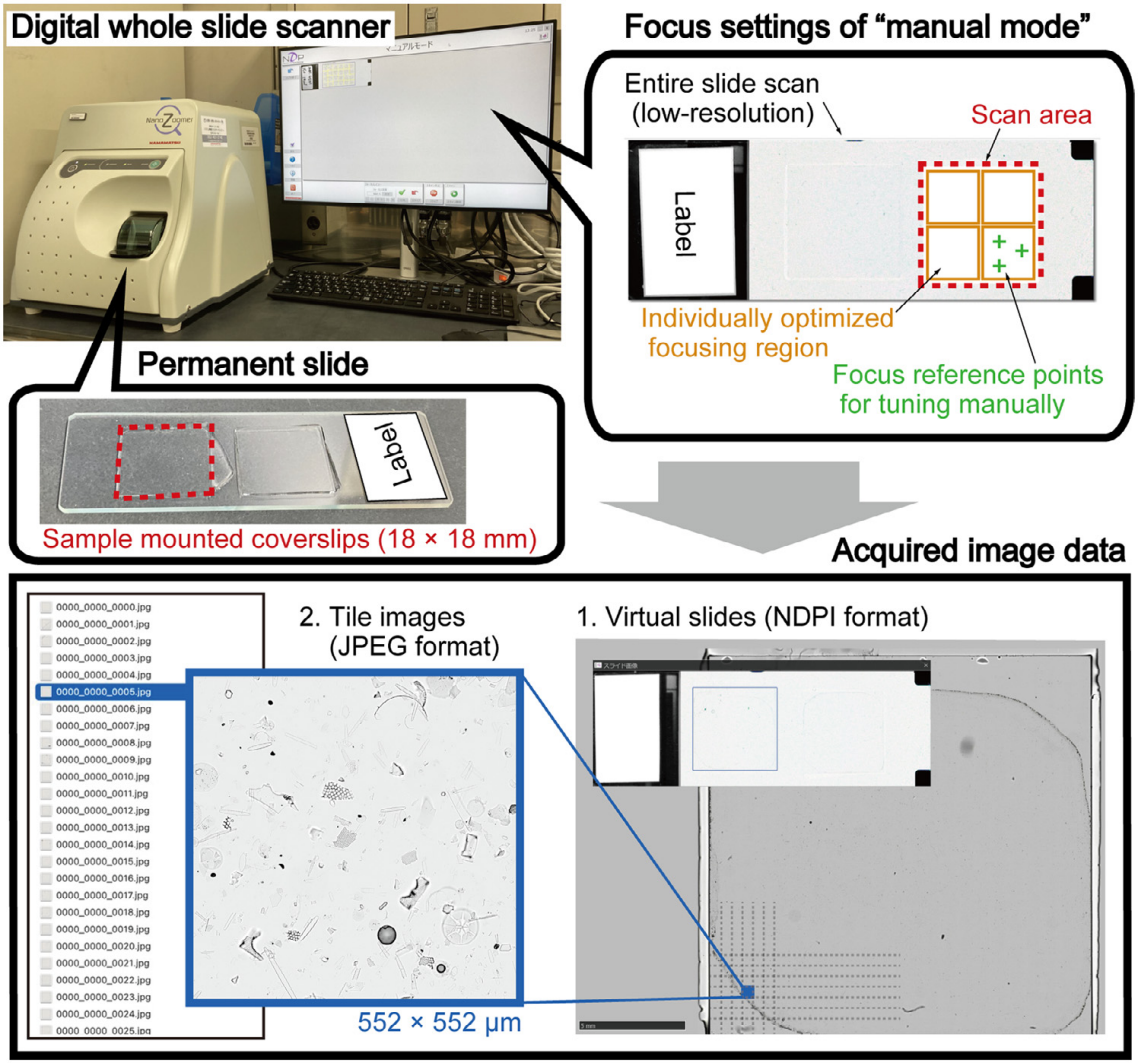
Overview of image acquisition from permanent slides using a slide scanner.

The virtual slides are transmitted light microscopy images of the coverslip on each permanent slide (Fig. 2). Each virtual slide is named according to the coverslip code of the permanent slide and is linked to the name of the tile images described in the following section. All virtual slides are provided in ndpi format. The detailed specifications of this format are not publicly available, but a brief description is provided by the OpenSlide project [9,10]. Browsing ndpi format files requires the software “NDP.view2” (free image-viewing software developed by Hamamatsu Photonics K.K.) or use of the software ImageJ with NDPITools plugins [11]. On the NDP.view2 software, changing the focus depth for each virtual slide is supported for more detailed observation at 0.23 μm/pixel resolution. Note that, because the total size of all virtual slides prepared for the tile images (see next paragraph) exceeds the storage capacity of the data repository, only a subset of scanned areas is provided here as examples. The information and imaging conditions used to generate the tile images are summarized in the csv file (virtualslides-scanning-infomations.csv), and the original virtual slides are available from the corresponding author upon request.

The tile images consist of field-of-view images (regions of 552 μm × 552 μm) converted by segmenting the virtual slide. Each tile image is named according to the prefix of the original virtual slide name followed by the *x, y*, and *z* coordinates of the segmented region. Only annotated tile images from the representative (intermediate) focal layer are stored for each sampling site.

The annotations are targeted at *Eucampia antarctica* (Castracane) Mangin, a diatom species endemic to the Southern Ocean. The annotation text files were prepared in accordance with the YOLO format, which includes the class number, center *x* and *y* coordinates, width, and height of each bounding box. Because this dataset deals with only one class (*E. antarctica*), the class number is set to 0 in all annotation files. Each text file corresponds to one tile image and records the locations of one or more bounding boxes. Therefore, the annotation files are named identically to their associated tile images. All coordinates, widths, and heights are expressed as relative values, assuming the tile image’s height and width are equal to 1.

Based on the annotated tile images described above, multiple YOLO models were trained, including those trained on data from a single site (Sites CD, St12B, MC10, and GC2003) and those trained on pairwise combinations of these sites. Please note that not all sampling sites provided in the dataset were used for training the models. Each training experiment is organized into separate folders named according to the site(s) used for training, which contain the trained model (best.pt). In addition, to ensure reproducibility of the experiments, the training configuration file (hyp.yaml) and the training results (results.csv) are also included.

## 4 Experimental Design, Materials and Methods

### 4.1 Sediment sample selection and permanent slide preparation

Permanent slides for light microscopy observation were prepared from the surface layer (0–2 cm) of 18 sediment cores (Fig. 1). The cores were collected mainly from the winter sea-ice coverage zone in the Indian sector of the Southern Ocean, except for one site, site MC10, from the Bransfield Basin in the Atlantic sector. These sampling sites encompass a range of geomorphic features, ranging from the continental shelf to the abyssal plain, but exclude the plateau area. The samples were obtained by the Technology Research Center of Japan National Oil Corporation (JNOC) in 1983–1999 using R/V *Hakurei-maru*; the National Institute of Polar Research (NIPR) in 2018–2020 using the icebreaker *Shirase*; and KH19–6-cruise scientists using R/V *Hakuho-maru*. All sediments were acquired using a grab sampler (sites with prefixes "G" and CD, St12B, and St83), a gravity corer (sites with prefix "GC"), or a multiple corer (sites with prefix "MC"). Therefore, all samples from the surface layer contain a modern microfossil assemblage. Biostratigraphic results for large-bore gravity cores have been documented in the cruise reports [12], confirming that geologically modern sediments are preserved in the uppermost layers of the cores. The detailed locations of all sites are summarized in a csv file (Tile images/sample-locations.csv).

Methods of sediment treatment and slide preparation followed mainly Renberg [13] and Schrader and Gersonde [14], respectively. Approximately 0.1–0.2 g of dried sediment was placed in a 200 mL beaker containing approximately 1 mL hydrogen peroxide (H_2_O_2_, 10 %) and hydrochloric acid (HCl, 10 %) and boiled to remove organic and calcareous materials. Distilled water was added to a volume of 200 mL and left for 5 h to separate the residues and acidic water. The residue was separated by decanting the supernatant, and the beaker was then refilled with distilled water. This process was repeated four times to neutralize the suspension. All processed deposits were then preserved in 4 or 13.5 mL stored bottles. Permanent slides were made from the deposits in the stored bottles. Approximately 2–20 μL of agitated suspension with 500 μL distilled water was settled and dried on a coverslip (NEO Micro Cover Glass, Matsunami Glass Industries Ltd., Osaka, Japan), and the coverslip was fixed onto a slide (MICRO SLIDE GLASS, S1214, Matsunami Glass Industries Ltd., Osaka, Japan) with mounting media (Norland optical adhesive No 61, refractive index: 1.56). We used coverslips 24 mm × 32 mm, 18 mm × 24 mm, and 18 mm × 18 mm in size. The size linked with each virtual slide and tile images is noted in a csv file (virtualslides-scanning-infomations.csv).

### 4.2 Imaging methods

Acquisition of virtual slides was performed with a NanoZoomer SQ (a digital whole slide scanner developed by Hamamatsu Photonics K.K., Fig. 2). All virtual slides were obtained with a 20× objective lens (N.A. 0.75) at 40× scanning speed mode. Scanning was conducted with the dedicated software "NDP.scan for SQ". After an initial low-resolution scan of the entire slide glass, adjustment of the focus plane was performed in “manual mode.” In this mode, an optimized focusing region and representative focus points were defined manually, and the focus plane at each point was adjusted either automatically or manually, depending on the condition. This configuration allows each optimized focusing region to form an independent focus plane based on manually tuned focus reference points. In this study, individually optimized focusing regions of approximately smaller than 10 mm × 10 mm were positioned adjacent to each other so as to collectively cover the entire coverslip (Fig. 2). In addition, three to four representative focus points were defined for each of these regions (Fig. 2). Based on empirical experience, this configuration ensured that as many diatom valves as possible across the entire coverslip were brought into focus, minimizing the need for manual fine-tuning. Even with an 18 mm × 18 mm coverslip, distortions in the mounted sample and mounting medium often cause significant defocusing across the entire area when a single focal plane is applied.

Multiple focus planes were captured per scan, with focal layers spaced at ±0.5–2 μm intervals from the reference plane (details in the file Tile images/virtualslides-scanning-infomation.csv). Setting multiple focus planes enables the virtual slide to be viewed with adjustable focus during observation. In this study, the spacing between focal planes was defined by considering the valve height of diatoms, allowing overlapping valves to be observed at different focal depths.

Tile images (2400 pixels × 2400 pixels, 552 μm × 552 μm) were generated from virtual slides using the dedicated software "NDP.toolkit". The tile images were assigned X and Y coordinates starting from the top left corner when the whole scanned area was segmented. “Edge padding” was enabled so that even when the regions located at the edges of the scanned area (i.e., the rightmost column and the bottom row) were divided into regions smaller than the specified size, the corresponding images were output at the specified size, with blank areas filled with a constant background color.

### 4.3 Preparation of annotations and YOLO object detection models

Annotations for object detection linked with tile images were performed using Visual Object Tagging Tool (v2.2.0, Microsoft). Annotations for detecting the diatom species *E. antarctica* were first saved temporarily in JSON format and then converted to YOLO format with Unix shell commands. One bounding box was assigned to each occurrence of one complete valve or one countable valve fragment. Countable valve fragments were defined as those in which one side of the basal valve margin and one apical elevation were observable. All annotation files include bounding boxes with one category of “*Eucampia antarctica*”, which were drawn by one of the authors (SI), a researcher with almost 10 years of experience working on polar diatom assemblage analysis.

All models provided in this dataset were trained using the included tile images and annotation text files with YOLOv5x (Ultralytics, version v7.0–284 g95ebf68f). The prepared images were split into 80 % for training and 20 % for validation. When images were selected from two sites, 225 images were randomly sampled from each site and used to construct a training dataset. All model training was executed on a local computing environment featuring an Intel Core i7–10,700 K CPU, 64 GB RAM, and an NVIDIA GeForce RTX 3090 GPU (24 GB VRAM) under Windows 10. Each training run was conducted for up to 200 epochs with a batch size of 16, and the best-performing model was selected based on its validation performance. Details of the hyperparameter settings used for each training case are provided in the file “hyp.yaml”.

## Limitations

The overall assemblage analysis using images in this dataset can lead to biases in the relative abundance of specific taxa or valve abundance. Because the tile images include only those capturing *E. antarctica*, counting valves within these images is likely to result in overrepresentation of this species. In addition, the permanent slides were prepared with the pipette method [15], and the virtual slides capture only part of the coverslips; therefore, they cannot represent the uneven distribution of particles on the entire coverslips [14]. As a result, valve abundance per area counted from the virtual slides may be overestimated compared with those obtained from counts along random traverse lines under the microscope. To avoid these biases, full-coverslip virtual slides or complete tile-image sets are preferred and can be obtained from the corresponding author.

The fine structures of small diatoms may not be clearly visible at the 0.23 μm/pixel resolution of the slide scanner. Small *Fragilariopsis* species that are characterized by dense transapical striae (>10/10 μm) and fine poroids (>30/10 μm) [16] occur in the samples. Although their overall outlines can be recognized in the tile images and virtual slides, their fine structural features cannot be reliably measured based on this dataset.

## Ethics Statement

The authors have read and follow the ethical requirements for publication in Data in Brief. This work does not involve human subjects, animal experiments, or data collected from social media platforms.

## CRediT Author Statement

**Saki Ishino:** Conceptualization, Data curation, Methodology, Visualization, Writing – original draft, Funding acquisition. **Takuya Itaki:** Resources, Writing – review & editing, Supervision, Funding acquisition.

## Data Availability

Mendeley DataFossil diatom microscopy image datasets and annotations for object detection (Original data)
